# Overexpression of *bla*_SHV-12_ caused by tandem amplification contributed to ceftazidime/avibactam resistance in hypervirulent and carbapenem-resistant *Klebsiella pneumoniae*

**DOI:** 10.1080/22221751.2024.2426481

**Published:** 2024-11-05

**Authors:** Chao Liu, Juan Yi, Ping Yang, Chunjing Du, Fan Jiang, Ming Lu, Pengcheng Du, Ning Shen

**Affiliations:** a Department of Infectious Disease, Peking University Third Hospital, Beijing, People’s Republic of China; b Center of Infectious Disease, Peking University Third Hospital, Beijing, People’s Republic of China; c Institute of Medical Technology, Peking University Health Science Center, Beijing, People’s Republic of China; d Department of Pulmonary and Critical Care Medicine, Peking University Third Hospital, Beijing, People’s Republic of China; e Qitan Technology Ltd., Chengdu, People’s Republic of China

**Keywords:** Hypervirulent *klebsiella pneumoniae*, ceftazidime/avibactam resistance, *bla*_SHV-12_, sequence type 11, gene amplification

## Abstract

We identified a novel ceftazidime/avibactam (CAZ/AVI) resistance mechanism in endemic sequence type 11 hypervirulent and carbapenem-resistant *Klebsiella pneumoniae* isolated from a patient who had not been exposed CAZ/AVI. Overexpression of *bla*_SHV-12_ caused by tandem gene amplification contributed to CAZ/AVI resistance instead of the carriage of *bla*_KPC-2_. Enhanced genomic surveillance is essential to identify emerging variants.

*Klebsiella pneumoniae* (Kp) is notorious for causing various infections and has evolved into the following two pathotypes: hypervirulent Kp (hvKp) and classical Kp (cKp) [1]. Sequence type 11 (ST11) Kp is an epidemic clone worldwide, especially in China, and poses a great threat to public health due to its stronger genome plasticity of antimicrobial resistance and/or virulence acquisition [2,3]. Recently, ST11 Kp has developed both hypervirulence and carbapenem resistance (CR-hvKp) worldwide, thereby becoming a vexing problem in clinical practice，especially during COVID-19 pandemic [4,5]. Douradinha et al. reported that CR-hvKp could represent ultravirulent or supervirulent phenotypes [6]. The combination of ceftazidime and avibactam (CAZ/AVI) has been regarded as a potential antimicrobial treatment for CR-hvKp [7,8]. However, CAZ/AVI resistant CRKP has been reported and is mostly caused by *bla*_KPC-2_ mutations [9]. Here, we characterized a clinical case with a patient who had not been exposed to CAZ/AVI, but one of the five ST11 CR-hvKp isolates from the patient exhibited CAZ/AVI resistance in vitro. We explored a novel CAZ/AVI resistance mechanism caused by tandem amplification of *bla*_SHV-12_ that emerged during in vivo evolution.

## Clinical case description

From 2017 to the present, we conducted active genomic surveillance of Kp among three hospitals in Beijing [10]. Among 151 isolates, we found five ST11 CRKP isolates that were successively isolated from a patient. The patient was 82 years old and presented with various underlying diseases. She suffered from severe pneumonia and was then admitted to the intensive care unit (ICU). She received latamoxef, cefoperazone/sulbactam, piperacillin/sulbactam and meropenem treatment before the index Kp isolation. Five Kp isolates (Kp1, Kp2, Kp2A, Kp2B, Kp3) were isolated successively during four days of hospitalization in the ICU. The first isolate (Kp1) was obtained in sputum on the tenth day and had resistances to carbapenems (Supplementary Table). Then, tigecycline was added for treatment. Two Kp isolates (isolate Kp2A and Kp2B) were obtained from blood culture two days later, respectively. Interestingly, we obtained a CZA resistant isolate Kp2 from catheter on the same day. The last isolate Kp3 was obtained from blood culture on the 13th day. The patient died nine days after the isolation of the index isolate.

## Phenotypic and genomic characterization of ST11 CR-hvKp

We further performed susceptibility testing to confirm the minimal inhibitory concentrations (MICs) of CAZ/AVI in these isolates. Notably, although the patient had not been exposed to CAZ/AVI before admission, Kp2 represented CAZ/AVI resistance (MIC = 16/4 mg/L), as was determined by using Etest, according to the CLSI guideline. It was different from the CAZ/AVI susceptibility profile of other four isolates (MIC = 12/4 mg/L), indicating within-host heterogeneity (Supplementary Table). The susceptibility profile of the five isolates against other tested antimicrobials were the same, except for imipenem-relebactam and ceftolozane-tazobactam (Supplementary Table). Based on the susceptibility profiles, of the four isolates that were not resistant to CAZ/AVI, the first isolate Kp1 was selected for subsequent experiments. According to the detection of virulence genes (*rmpA2 *+ *iucA*) [11] and the hypervirulent phenotypes determined by the *Galleria mellonella* infection model (Supplementary Figure 1A), the isolates Kp1 and Kp2 were both identified as CR-hvKp. In addition, both two isolates showed the production of siderophore compared with strain NTUH-K2044, possessed the capacity to form biofilms in vitro and had no significant difference in fitness costs (Supplementary Figure 1).

We further obtained the complete genome sequences of the five isolates by using a combination of a next-generation sequencing platform (Illumina, USA) and a nanopore long-read sequencing platform (QitanTech, China). Sequence analysis revealed that the five isolates belonged to ST11, and the capsule type was KL47, as determined by *Kleborate* software [12]. Only two single nucleotide polymorphisms (SNPs) were identified in the chromosomal sequences, suggesting that they were nearly identical. Additionally, the SNPs were located in the intergenic region and not associated with any genes related to CAZ/AVI resistance, including the genes encoding KPC and outer membrane proteins.

## Tandem amplification and overexpression of plasmid-borne *bla*_SHV-12_ in the CAZ/AVI resistant isolate

**Figure 1.**
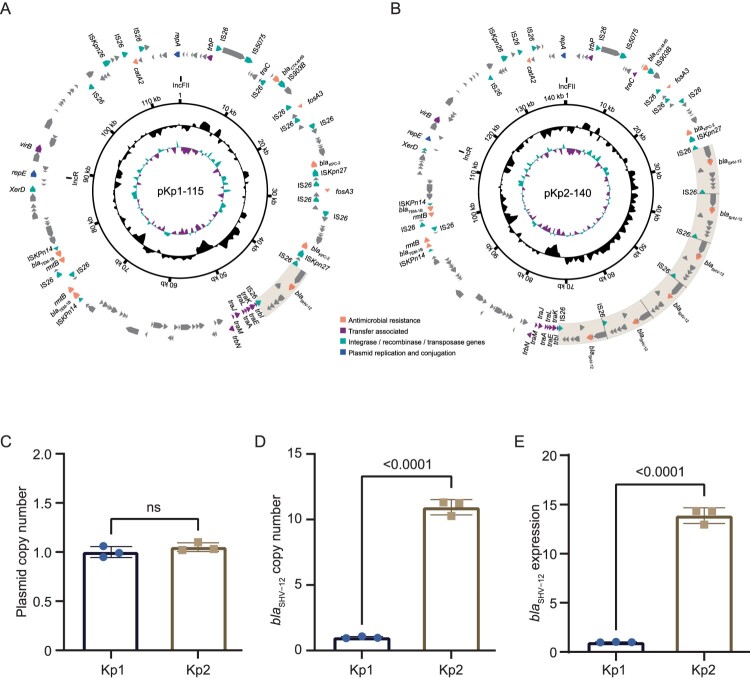
Variations in *bla*_SHV-12_ bearing IncR/IncFII plasmids. (A) and (B) Circular sketch map of the two plasmids. The rings from inner to outer represent the GC skews, GC content, genome scales and predicted open reading frames. The arrows of the two outer rings represent the genes related to resistance and transfer (red: antimicrobial resistance genes; green: integrase recombinase and transposase genes; purple: transfer associated genes; dark blue: plasmid replication related genes; and grey: genes of other functions). The 7.3-kb fragments encoding *bla*_SHV-12_ are in light brown. (C–E) The copy number of *bla*_SHV-12_ bearing the IncR/IncFII plasmid, the copy number of *bla*_SHV-12_, and the relative expression level among the Kp1 and Kp2.

We further analysed the plasmid profiles of the isolates. All isolates harboured a 109-kb IncFIB plasmid, which were identical without any SNPs and indels and did not encode any resistance genes. All isolates contained similar multireplicon IncR/IncFII plasmids, of different sizes, harbouring resistance genes, including *bla*_KPC-2_, *bla*_TEM-1B_, *bla*_SHV-12_ and *bla*_CTX-M-65_ (Figure 1A,B, Supplementary Figure 2). Interestingly, the IncR/IncFII plasmids of CAZ-AVI susceptible isolates all harboured only one copy of *bla*_SHV-12_, whereas the plasmid pKp2-140 of the CAZ-AVI resistant isolate Kp2 harboured 6 copies of *bla*_SHV-12_. Meanwhile, these *bla*_SHV-12_ copies were all located in the nearly identical 7.3-kb fragments flanked by IS*26*, and the six copies formed a tandem repeat structure in pKp2-140 (Figure 1A,B). Additionally, plasmid pKp1-115 of Kp1 also harboured two copies of *bla*_KPC-2_ and *bla*_TEM-1B_, which conferred meropenem and piperacillin/tazobactam resistance. However, plasmid pKp2-140 of Kp2 carried only one copy of *bla*_KPC-2_ and *bla*_TEM-1B_ but had CAZ/AVI resistance, suggesting that tandem amplification of *bla*_KPC-2_ and *bla*_TEM-1B_ might not be associated with CAZ/AVI resistance. The other parts of the plasmids were nearly identical.

We then performed qPCR assays to confirm the increases of the copy number and expression of *bla*_SHV-12_ in the CAZ/AVI-resistant isolate. First, compared to the single copy housekeeping gene *rpoB* in the chromosome, the relative copy number of *virB*, a single copy gene in the IncFII/IncR plasmids, was approximately 1 in both isolates, indicating that the copy number of the plasmids did not vary significantly (Figure 1C). In contrast, the relative copy number of *bla*_SHV-12_ was nearly 1 compared with *virB* in Kp1, but 11 in Kp2 (Figure 1D). Additionally, the reverse transcription qPCR of the mRNA of *bla*_SHV-12_ also revealed high expression of *bla*_SHV-12_ in Kp2, with a 14-fold change compared to that in Kp1 (Figure 1E). Therefore, we speculated that the tandem amplification of *bla*_SHV-12_ might be related to CAZ/AVI resistance.

## Discussion

ST11 is predominant in worldwide and ST11 strains are notorious for acquiring various resistance and virulence genes [2,3,13]; thus, these strains have a greater infectious burden in high-risk areas of CR-hvKp [1]. Epidemiological and genomic researches in China reported that *bla*_KPC-2_ is the main cause of CRKP [13] and emphasized that CAZ/AVI might be an appropriate option for CRKP [7,8]. In this study, we suggested that the tandem amplification and overexpression of *bla*_SHV-12_ in an IncFII/IncR plasmid might be the reason for the elevation of MIC and the emergence of CAZ/AVI resistance among ST11 CR-hvKp strains during in vivo evolution. Notably, compared to the isolates with only one copy of *bla*_SHV-12_, the CAZ/AVI-resistant isolate with six copies of *bla*_SHV-12_ did not represent a significantly different fitness cost, which might be the reason for its reservation without CAZ/AVI exposure.

Recently, CAZ/AVI-resistant Kp has been increasingly reported, which is a great challenge in developing effective anti-infective treatments [7]. Most of the reported mechanisms have been associated with mutations in *bla*_KPC_, and various subgenotypes of *bla*_KPC-like_, including *bla*_KPC-14_, *bla*_KPC-23_ and *bla*_KPC-36_, were subsequently found to be correlated with CAZ/AVI resistance, even in cases with patients that were previously unexposed to CAZ/AVI [14]. These emerging *bla*_KPC_ variants have become new threat to global public health [15], however, the variations causing overexpression of known genes and subtypes might be more inconspicuous and noteworthy. Under ceftazidime selective pressure in vitro, an elevated *bla*_KPC-2_ copy number contributes to low-level CAZ/AVI resistance, whereas the mutation of *bla*_KPC-2_ results in high-level resistance [16]. Other *β* – lactamases, such as VEB-25, were also reported leading to the outbreak of CAZ/AVI-resistant Kp [17]. Recently, after CAZ/AVI antimicrobial treatment, Cui et al. reported that genetic rearrangement of plasmids and chromosomes led to the formation of an additional promoter for *bla*_SHV-12_, resulting in the overexpression of *bla*_SHV-12_ and subsequently leading to CAZ/AVI resistance [18]. Here, we reported a novel mechanism by which CAZ/AVI resistance could be caused by *bla*_SHV-12_ amplification and overexpression during in vivo evolution. These indicate that the necessity of developing vaccines and other immunotherapies to combat antimicrobial resistance, and the development of novel antimicrobials is not enough, as the emergence of resistances to new drugs seems to be faster and faster [19–21]. Importantly, this variation had no significant fitness cost, underlining the importance of the continuous surveillance of genomic trends and the identification of emerging variants.

## Conclusion

Within-host evolution caused by *bla*_SHV-12_ amplification resulted in *bla*_SHV-12_ overexpression and CAZ/AVI resistance among CR-hvKp strains, which is a novel mechanism reported in patients who had not been exposed to CAZ/AVI. By using real-time long-read sequencing, such as nanopore technology, this kind of variation could be well studied and should be enhanced in clinical practice.

## Supplementary Material

Supplementary_Figure2.eps

Supplementary_Materials_clean.docx

Supplementary_Figure1.eps

## Data Availability

The genome sequences in this study were deposited in the NCBI database under BioProject accession numbers PRJNA972494.
