# Supplementary material for: Overexpression of blaSHV-12 caused by tandem amplification contributed to ceftazidime/avibactam resistance in hypervirulent and carbapenem-resistant Klebsiella pneumoniae
Source: Emerg Microbes Infect. 2024 Nov 5;13(1):2426481. doi: 10.1080/22221751.2024.2426481 (PMC11565672; doi:10.1080/22221751.2024.2426481)
Supplement: Supplementary_Materials_clean.docx [file TEMI_A_2426481_SM6358.docx]

**Supplementary** **Materials**

**Supplementary Table**. Antimicrobial susceptibility testing results for the five ST11 CR-hvKp

| Items | Kp1 | Kp2 | Kp2A | Kp2B | Kp3 |
| --- | --- | --- | --- | --- | --- |
| Amikacin | ≥64 | ≥64 | ≥64 | ≥64 | ≥64 |
| Tobramycin | ≥16 | ≥16 | ≥16 | ≥16 | ≥16 |
| Aztreonam | ≥64 | ≥64 | ≥64 | ≥64 | ≥64 |
| Levofloxacin | ≥8 | ≥8 | ≥8 | ≥8 | ≥8 |
| Ciprofloxacin | ≥4 | ≥4 | ≥4 | ≥4 | ≥4 |
| Ceftriaxone^b^ (mm) | 6 | 6 | 6 | 6 | 6 |
| Cefepime | ≥32 | ≥32 | ≥32 | ≥32 | ≥32 |
| Ceftazidime | ≥64 | ≥64 | ≥64 | ≥64 | ≥64 |
| Ceftazidime-avibactam^c^ | 12/4 | 16/4 | 12/4 | 12/4 | 12/4 |
| Cefoperazone-sulbactam | ≥64 | ≥64 | ≥64 | ≥64 | ≥64 |
| Piperacillin-tazobactam | ≥128 | ≥128 | ≥128 | ≥128 | ≥128 |
| Imipenem | ≥16 | ≥16 | ≥16 | ≥16 | ≥16 |
| Meropenem | ≥16 | ≥16 | ≥16 | ≥16 | ≥16 |
| Ertapenem^b^ (mm) | 6 | 6 | 6 | 6 | 6 |
| Doxycycline | 4 | 4 | 4 | 4 | 4 |
| Minocycline | 8 | 8 | 8 | 8 | 8 |
| Tigecycline ^c^ | 0.38 | 0.38 | 0.38 | 0.38 | 0.38 |
| Imipenem-relebactam^c^ | 0.75 | 0.75 | 0.75 | 1.5 | 1 |
| Ceftolozane-tazobactam^c^ | 64 | >256 | 64 | 64 | 64 |

a: The breakpoint was interpreted by the Clinical and Laboratory Standards Institute (CLSI) guidelines; b: The CRO and ETP was evaluated by the K-B method；c: Ceftazidime-avibactam, tigecycline, imipenem-relebactam and ceftolozane-tazobactam was assessed by the Etest.

**Supplementary Figure 1**. Virulence related phenotype results for the Kp1 and Kp2. A) Virulence in *Galleria mellonella* model of the enrolled Kp isolates. NTUH-K2044 as positive control. B) Siderophore production of the enrolled Kp isolates. C) Biofirm production of the enrolled Kp isolates. D) Growth curves of the enrolled Kp isolates.

**Supplementary Figure 2**. Circular sketch map of the IncR/IncFII plasmids of Kp2A, Kp2B, and Kp3. The rings from inner to outer represent the GC skews, GC content, genome scales and predicted ORFs. The arrows of the two outer rings represent the genes related to resistance and transfer (red: antimicrobial resistance; green: integrase recombinase and transposase genes; purple: transfer associated; dark blue: plasmid replication; and gray: other functions)
